# Changes in Symbiotic Microbiota and Immune Responses in Early Development Stages of *Rapana venosa* (Valenciennes, 1846) Provide Insights Into Immune System Development in Gastropods

**DOI:** 10.3389/fmicb.2020.01265

**Published:** 2020-06-16

**Authors:** Mei-Jie Yang, Hao Song, Zheng-Lin Yu, Zhi Hu, Cong Zhou, Xiao-Long Wang, Tao Zhang

**Affiliations:** ^1^CAS Key Laboratory of Marine Ecology and Environmental Sciences, Institute of Oceanology, Chinese Academy of Sciences, Qingdao, China; ^2^Laboratory for Marine Science and Technology, Qingdao National Laboratory for Marine Science and Technology, Qingdao, China; ^3^Center for Ocean Mega-Science, Chinese Academy of Sciences, Qingdao, China; ^4^CAS Engineering Laboratory for Marine Ranching, Institute of Oceanology, Chinese Academy of Sciences, Qingdao, China; ^5^University of Chinese Academy of Sciences, Beijing, China

**Keywords:** *Rapana venosa*, symbiotic microbiota, immune system, metamorphosis, early development stages

## Abstract

The symbiotic microbiota can stimulate modulation of immune system, which also can promote immune system mature in critical developmental periods. In this study, we have investigated the symbiotic microbiota in *Rapana venosa* at five early development stages using Illumina high-throughput sequencing, and detected immune responses in larvae. Analysis of the symbiotic microbiota sequences identified that the most abundant phylum was Proteobacteria. Beta diversity analysis indicated that the structure of the symbiotic microbiota dramatically shifted in early development stages. The abundance of immune-related KEGG Orthologs (KOs) also increased in competent larval (J4, 30-day post-hatching) and postlarval after 3 days of metamorphosis (Y5, 33-day post-hatching) stages. Acid phosphatase activity decreased significantly in the Y5 stage, and alkaline phosphatase activity also at a lower level in Y5 stage, whereas lysozyme activities exhibited no remarkable change. Also, the activities of catalase and superoxide dismutase activities decreased dramatically during early development stages of *R. venosa.* Dramatic changes in the symbiotic microbiota and the immune response mainly occurred in the initially hatched veliger (C1), competent larval (J4) and postlarval (Y5) stages, during which the hosts might experience substantial environmental changes or changes in physiological structure and function. These findings expand our understanding of the stage-specific symbiotic microbiota in *R. venosa* and the close association between immune system and symbiotic microbiota in mollusks, however, the specific relationship may need more researches are needed to investigated in the future.

## Introduction

The symbiotic microbiota is a complex ecosystem with multiple functions significant for the health of host (Ramirez and Romero, 2017), and the intricate interactions between the symbiotic microbiota and host start from early development stages in host (Nicholson et al., 2012). During host development, the symbiotic microbiota serves as important roles, including harvesting energy, against pathogens, and stimulating the development of the immune system (Al-Harbi and Uddin, 2005; Woodhams et al., 2007; Buchon et al., 2013; Ramirez and Romero, 2017). Furthermore, when the host undergoes a critical developmental event, corresponding changes also occur in its symbiotic microbiota that can reflect changes in the physiological functions of the host (Fraune and Bosch, 2010; Li et al., 2017), especially the immune system.

The veined rapa whelk (*Rapana venosa*) is an important fishery resource in China, and native to temperate Asian waters (Mann and Harding, 2003). Recently, the population of *R. venosa* has dramatically decreased because of the overexploitation and destruction of breeding grounds (Wei and Wang, 1999). Although efforts involving in the commercial aquaculture of *R. venosa* have been measured by enterprises since 1992 (Yuan, 1992), large-scale cultivation has been still severely restricted by the high mortality in larvae during metamorphosis (Song et al., 2017b). Metamorphosis is one of the most important developmental events in the life cycle of mollusks, usually accompanied by a high mortality (Harding, 2006). Furthermore, many morphological and behavioral variation occur during metamorphosis, including velum degradation and reabsorption, foot proliferation and elongation, and initiation of rapid secondary shell growth (Song et al., 2017b), all of which are regulated by the nervous, digestive and immune systems. Correspondingly, many changes occur in these systems at the molecular level during this important developmental process (He et al., 2014; Ueda and Degnan, 2014; Song et al., 2016b; Shen et al., 2018).

There are many studies about early development stages of *R. venosa* (Harding, 2006; Hacer and Ertug, 2007; Pan et al., 2013; Ban et al., 2014), and recent studies based on omics have paid more attention to the regulatory mechanism of its metamorphosis (Song et al., 2016a,b,c,d, 2017a,b). Additionally, a previous study explored the neuronal development of early larvae of *R. venosa* and investigated larval metamorphosis and feed conversion in *R. venosa* (Pan, 2014; Yang et al., 2016), suggesting that changes occur in the digestive system during the early development stages. Whereas, few studies investigated the development of immune system in *R. venosa* during metamorphosis, although the immune system is significant in early developmental stages, which has been extensively studied in other mollusks (Bao et al., 2015; Shen et al., 2018).

Accumulating evidence has shown that the symbiotic microbiota can stimulate the modulation of the immune system, resulting in mature and balanced immune responses (Mazmanian et al., 2005; Edelman et al., 2008). The effects are often reflected in the changes of immune enzymes [acid phosphatase (ACP), alkaline phosphatase (ALP), lysozyme (LSZ), superoxide dismutase (SOD), catalase (CAT) (Canicatti, 1990; Janeway and Medzhitov, 2002; Song et al., 2010)] and immune-related genes [Toll-like receptor 2, tumor necrosis factor, and defensin genes (Song et al., 2016b)]. Therefore, there is a close relationship between assembly of symbiotic microbiota and the development of host immune system. During early research, many studies examined the symbiotic microbiota of vertebrates such as rats and fishes (Mazmanian et al., 2005; Dhanasiri et al., 2011; Stephens et al., 2015; Li et al., 2017), while in recent years, due to experimental advantages, invertebrates have been used to help us understand the microbe–host interactions (Graf, 2016). However, few studies have focused on the symbiotic microbiota during the development of mollusks, and only Zhao et al. (2012) described the composition of intestinal symbiotic microbiota at different development stages of abalone. The changes that occur in the symbiotic microbiota composition during the development of mollusks may reflect the development of the immune system, which may play a regulatory role in metamorphosis.

In the present study, we aimed to elucidate the structure changes of the symbiotic microbiota during early development stages of *R. venosa*. and investigate the relationship between the development of the immune system and symbiotic microbiota. This information could help to expound the correlations between symbiotic microbiota and the development of the immune system, as well as further understand the mechanism of metamorphosis in *R. venosa*.

## Materials and Methods

### Sample Collection

Culturation of larval rearing was conducted according to Song et al. (2016b). Planktonic larvae were feed with a mixture of *Isochrysis galbana, Chlorella vulgaris*, and *Platymonas subcordiformis* three times a day. Samples were collected from five early developmental stages: the one-spiral whorl stage [C1, 1 day post-hatching (dph)], two-spiral whorl stage (D2, 5 dph), three-spiral whorl stage (F3, 12 dph), four-spiral whorl stage (competent larva, J4, 30 dph) and postlarval stage after 3 days of metamorphosis (Y5, 33 dph) (Pan et al., 2013; Song et al., 2016c). Each developmental stage had five replicates (30 larvae were used in each replicate). Larvae in each collected sample were euthanized and sterilized with 70% ethanol for 1 min and then washed three times using sterile water, snap-frozen and preserved at −80°C for DNA extraction. Environmental samples included five bait samples and five water samples. Five hundred milliliters of food samples and water samples were filtered through 0.22 μm cellulose nitrate filter, then subjected to bead beating and DNA extraction from the filter using the same method used for the other samples.

The samples from each development stage were homogenized and used to extract genomic DNA, total protein and total RNA by using the DNA/RNA/Protein Coextraction Kit (DP423, TIANGEN Biotech Co., Ltd., Beijing, China) according to the manufacturer’s protocol, and the extracted products were used for PCR amplification, analysis of immune enzyme activity and qRT-PCR, respectively. Subsequently, the DNA and RNA concentrations were detected by a NanoDrop 1000 (Thermo Fisher Scientific, United States), and the purities were analyzed according to the ratio of the absorbance values at 260 and 280 nm (OD 260/OD 280).

All procedures involved in the animal collection, rearing and dissection were conducted following the Guideline of Ethical Regulations of Animal Welfare of the Institute of Oceanology, Chinese Academy of Sciences (IOCAS 2013.3). Our study protocols were approved by the Animal Welfare Committee of the IOCAS.

### PCR Amplification and Illumina MiSeq Platform Sequencing

PCR amplification of 16S rRNA gene and sequencing were performed by Majorbio Co., Ltd. (Shanghai, China). PCR amplification was carried out using the 338F/806R primer targeting V3-V4 regions of the bacterial 16S rRNA gene (338F 5′-ACTCCTACGGGAGGCAGCAG-3′ and 806R 5′-GGACTACHVGGGTWTCTAAT-3′) (Chu et al., 2015). PCR products were purified by the AxyPrep DNA Gel Extraction Kit (Axygen Biosciences, Union City, CA, United States), and quantified by QuantiFluor^TM^-ST (Promega, United States), then sequenced (2 × 250 bp) on the MiSeq PE300 platform (Illumina, United States).

### Analysis of Immune-Related Enzyme Activity

The soluble protein content of each samples was detected using Bradford (1976) method and expressed as mg mL^–1^ of larval serum. ACP and ALP were determined according to Barrett (1972) (A060-1-1, A059-1-1), and a unit of enzyme activity was defined as the amount of enzyme required to produce 1 μmol of phenol, and expressed as ACP/ALP King unit mL^–1^. LSZ activity was defined 7776 in terms of the change in absorbance of a *Micrococcus luteus* cell suspension per minute and expressed as LSZ unit mL^–1^. SOD activity was analyzed according to Ôyanagui (1984). A unit of SOD activity was defined as the amount of enzyme inhibited superoxide-induced oxidation by 50%, and expressed as SOD unit mL^–1^. CAT activity was determined according to Góth (1991) (A007-1-1). A unit of CAT activity was defined as the amount that can catalyze the conversion of 1 μmol H_2_O_2_ s^–1^, and expressed as CAT unit mL^–1^. All immune enzyme activities were measured following the manufacturer’s protocols of assay kits (Nanjing Jiancheng Bioengineering Institute, China).

### Gene Expression Analysis

Immune-related gene expression was determined at different development stages. First-strand cDNAs for qRT-PCR were synthesized by a Prime Script^TM^ RT Reagent Kit with gDNA Eraser (TaKaRa, Japan). Primers used for gene expression analysis were designed according to the full-length cDNA sequences of Toll-like receptor 2, tumor necrosis factor and defensin genes (Supplementary Table S1), the levels of which dramatically shifted during metamorphosis, which may be associated with the development of the immune system (Song et al., 2016b). The gene encoding the 60S ribosomal protein L28 (RL28-F, RL28-R) was selected as the housekeeping gene for signal normalization, which is the most stable gene in all developmental stages in *R. venosa*. (Song et al., 2017a). SYBR PrimeScript^TM^ RT-PCR Kit II (TaKaRa, Japan) was used with an Eppendorf Mastercycler^®^ ep Realplex (Eppendorf, Hamburg, Germany) for qRT-PCR analysis following the manufacturer’s protocols (Yang et al., 2020). Standard curves were made with 10-, 10^2^-, 10^3^-, 10^4^-, and 10^5^-fold dilutions of each cDNA template, and qRT-PCR was carried out with this program: 95°C for 5 s and 40 cycles of 95°C for 15 s, then 60°C (Toll-like receptor 2, defensin) or 58°C (tumor necrosis factor) for 30 s. The 2^–ΔΔCt^ method was used to analyzed the relative gene expression.

### Data Analysis

USEARCH 7.1^1^ (Caporaso et al., 2012) was used to demultiplex^2^ and quality filter raw sequences^3^ to discard singletons from 35 samples, and remove chimeric sequences^4^ to get clean reads (Haas et al., 2011). Operational taxonomic units (OTUs) were identified (equivalent to 97% sequence similarity) and clustered using UPARSE 7.1 software^1^ (Edgar, 2013). Meanwhile, effective sequences were aligned against the SILVA database^5^, and identified on phylum, class, order, family, genus and species levels by the Ribosomal Database Project Bayesian classifier^6^ with a 70% threshold. Partial data analysis was performed on the free online platform of the Majorbio Cloud Platform^7^.

OTU abundance information was normalized (Yang et al., 2019) for subsequent analysis. Alpha diversity indices used to assess the community diversity (Shannon and Simpson) and richness (Chao and ACE) were analyzed by Mothur^8^. Venn diagram analysis was performed using the Draw Venn Diagram online tool^9^ (Wang et al., 2019). Histograms of the community composition were conducted by the Vegan package in R 2.4^10^. Beta diversity analyses, including non-metric multidimensional scaling (NMDS), typing analysis and hierarchical clustering analysis, were performed using QIIME 1.7.0^11^ based on weighted UniFrac distances further investigated the similarity and differences of microbiota among samples from different development stages. In order to assess differences in the microbiota communities among groups, Adonis and ANOSIM were performed using the Vegan package in R 2.4. Permutational multivariate analysis of variance (PERMANOVA) was performed to compare dissimilarities in the structure of the microbiota (Anderson, 2001).

Microbial taxa that differed between every pair of groups at genera level were analyzed using linear discriminant analysis (LDA) effect size (LEfSe) method^12^, and Kruskal–Wallis test was used to identify the significant differences between two groups (Segata et al., 2011). Phylogenetic Investigation of Communities by Reconstruction of Unobserved States (PICRUSt) was used to predict the functions of microbiota by predicting the Kyoto Encyclopedia of Genes and Genomes (KEGG) categories and functional enzymes (Langille et al., 2013).

In present study, Levene’s test was used to determine the homogeneity of variances in analysis of immune-related enzyme activity and gene expression, and mean differences were determined using Student’s *t*-test. SPSS 19.0 was used in all statistical analysis, and *P*-value < 0.05 indicated statistically significant difference.

## Results

### Composition of the Symbiotic Microbiota Population

A total of 1,432,056 high-quality sequences were produced from 32 samples (J4_4, Y5_4 and Y5_5 were excluded from sequencing because of the low quality DNA): 956,119 from the host corresponding to the five culture stages and 475,937 from 10 environmental water and bait samples, with an average of 44,752 reads (Table 1). The data has been submitted to the sequence read archive (SRA) with the project accession number PRJNA574861. Clustered at 3% distance, a total of 1,879 OTUs were obtained with an average of 535 OTUs, and 1744 OTUs were from the host (Table 1).

**TABLE 1:** Sequencing and OTU classification information.

Sample name	Read number	Phylum	Class	Order	Family	Genus	Species	OTU
C1_1	44164	19	41	91	167	296	418	745
C1_2	39329	21	37	87	178	293	412	722
C1_3	34000	19	36	85	159	266	367	682
C1_4	55209	20	41	95	180	306	440	818
C1_5	42697	21	39	86	156	265	366	668
D2_1	32998	16	34	79	147	241	332	595
D2_2	37316	17	33	76	153	243	343	599
D2_3	34110	15	33	73	143	244	349	650
D2_4	45060	18	39	82	159	273	383	704
D2_5	33789	15	31	70	134	234	333	622
F3_1	48305	20	35	80	143	229	306	590
F3_2	35057	19	32	76	130	199	275	533
F3_3	31639	16	33	84	142	214	273	458
F3_4	40295	22	38	88	155	256	353	621
F3_5	41524	19	29	76	139	237	318	570
J4_1	44885	17	35	73	130	208	280	431
J4_2	50766	23	40	89	174	277	387	647
J4_3	36937	18	30	67	116	183	237	371
J4_5	38192	18	30	70	119	189	260	410
Y5_1	44057	21	43	99	166	285	409	649
Y5_2	42979	19	37	92	159	277	395	619
Y5_3	50920	20	42	97	167	280	391	639
F_1	34726	8	18	38	55	90	103	128
F_2	41223	8	17	45	61	107	124	154
F_3	46941	8	17	40	58	97	105	130
F_4	33010	7	15	32	49	78	88	107
F_5	29588	8	17	37	53	90	100	122
H_1	58530	25	50	103	176	308	470	658
H_2	34336	23	44	85	150	281	420	593
H_3	45501	24	47	89	160	292	450	633
H_4	42937	23	46	92	165	302	457	628
H_5	37707	25	47	92	166	293	447	637

*Summary of sequencing read analysis, numbers of OTUs, and numbers of OTUs that can be classified at different levels (phylum, class, order, family, and genus). C1, one-spiral whorl stage; D2, two-spiral whorl stage; F3, three-spiral whorl stage; J4, four-spiral whorl stage (competent larvae); Y5, postlarval stage after 3 days of metamorphosis; F, food and H, water.*

**FIGURE 1:**
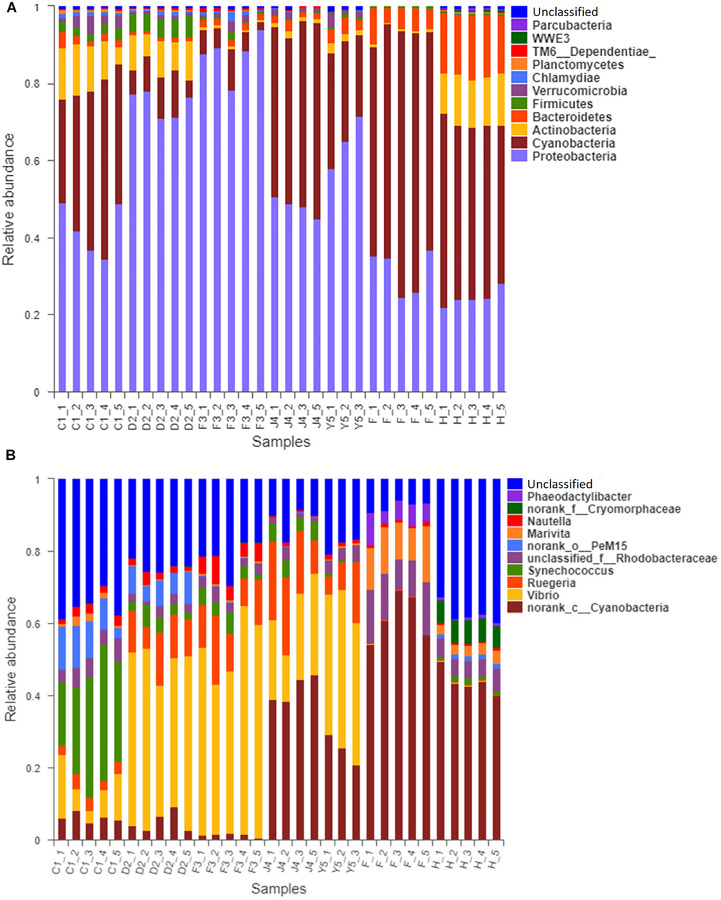
Relative read abundances of different bacterial phyla **(A)** and genera **(B)** within the different communities. Sequences that could not be classified into any known group are designated ‘unclassified.’ C1, one-spiral whorl stage; D2, two-spiral whorl stage; F3, three-spiral whorl stage; J4, four-spiral whorl stage (competent larvae); Y5, postlarval stage after 3 days of settling; F, food; H, water.

**FIGURE 2:**
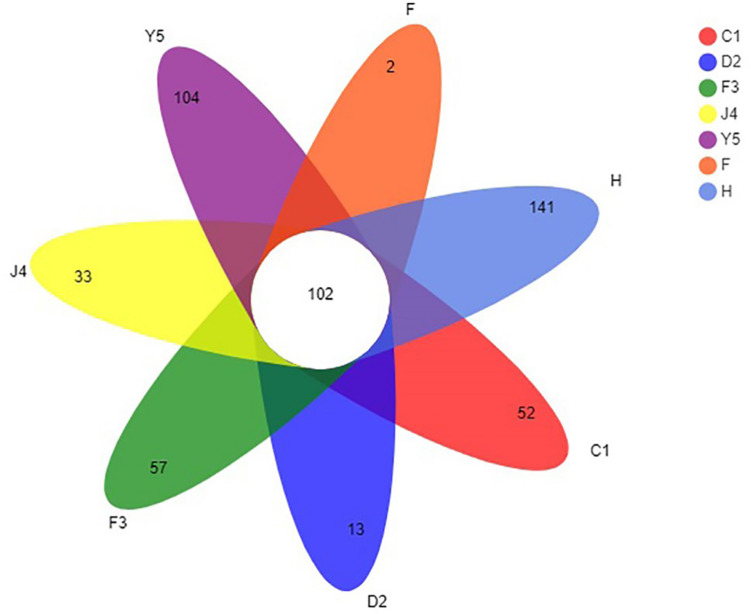
Venn diagram of OTUs in groups C1, D2, F3, J4, Y5, F, and H. The overlapping and separate sections represent the number of shared and unique OTUs, respectively. C1, one-spiral whorl stage; D2, two-spiral whorl stage; F3, three-spiral whorl stage; J4, four-spiral whorl stage (competent larvae); Y5, postlarval stage after 3 days of metamorphosis; F, food; H, water.

The OTUs were classified into 32 phyla. Sequences that was not classified into any known groups were assigned as ‘unclassified’ (unclassified < 0.005). The phyla with the highest relative abundance in all samples were Proteobacteria (54.02%), Cyanobacteria (32.41%), Actinobacteria (4.86%), Bacteroidetes (5.29%), Firmicutes (1.28%), Verrucomicrobia (0.84%), Chlamydiae (0.36%), Planctomycetes (0.20%), TM6_Dependentiae_ (0.23%), WWE3 (0.09%), and Parcubacteria (0.08%) (Figure 1A). Proteobacteria was the most abundant phylum among all 22 samples from the host except samples J4_3 and J4_5, in which Cyanobacteria was the most abundant phylum, and Cyanobacteria was also the most abundant phylum among the 10 samples from the environment. At the genus level, a total of 289 taxa were identified, and the most abundant genera were norank_c__*Cyanobacteria* (29.26%), *Vibrio* (25.23%), *Ruegeria* (7.95%), unclassified_f__*Rhodobacteraceae* (4.75%), *Synechococcus* (2.91%), *Marivita* (2.68%), norank_o__PeM15 (1.82%), *Nautella* (1.74%), norank_f__*Cryomorphaceae* (1.24%), and *Phaeodactylibacter* (1.07%) (Figure 1B). Additionally, Venn diagram constructed to identify dominant OTUs present in samples suggested that 102 OTUs were shared among the five stages and environment samples, and 52, 13, 57, 33, 104, 2, and 141 OTUs were unique to groups C1, D2, F3, J4, Y5, F and H respectively (Figure 2).

### Diversity Analysis of the Five Development Stages

**FIGURE 3:**
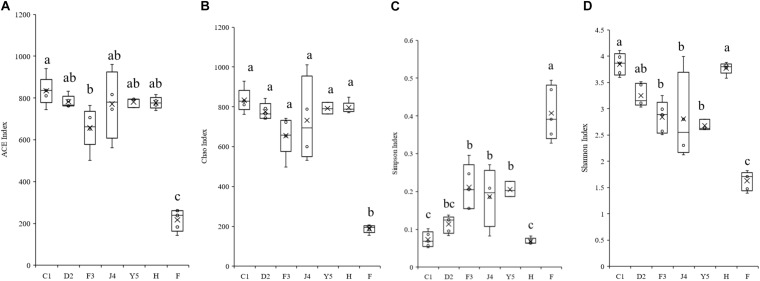
Boxplots showing the ranges of different alpha diversity indices. The boxplots show significant differences in the ACE index **(A)**, Chao index **(B)**, Simpson index **(C)**, and Shannon index **(D)** at different culture stages. Different lower-case letters for each bar indicate significant differences (*P* < 0.05). C1, one-spiral whorl stage; D2, two-spiral whorl stage; F3, three-spiral whorl stage; J4, four-spiral whorl stage (competent larvae); Y5, postlarval stage after 3 days of metamorphosis; F, food; H, water.

The alpha diversity of the microbiota community varied significantly in different development stages (Figure 3). The Shannon index ranged from 2.118 to 4.104 and significantly decreased with host development, while the Simpson index ranged from 0.052 to 0.494 and increased with host development, which suggests that the diversity of the symbiotic microbiota decreased with host development. The Chao index ranged from 155 to 929, while the ACE index ranged from 142 to 940, and both the Chao and ACE index did not show significant change with the development of the host.

**FIGURE 4:**
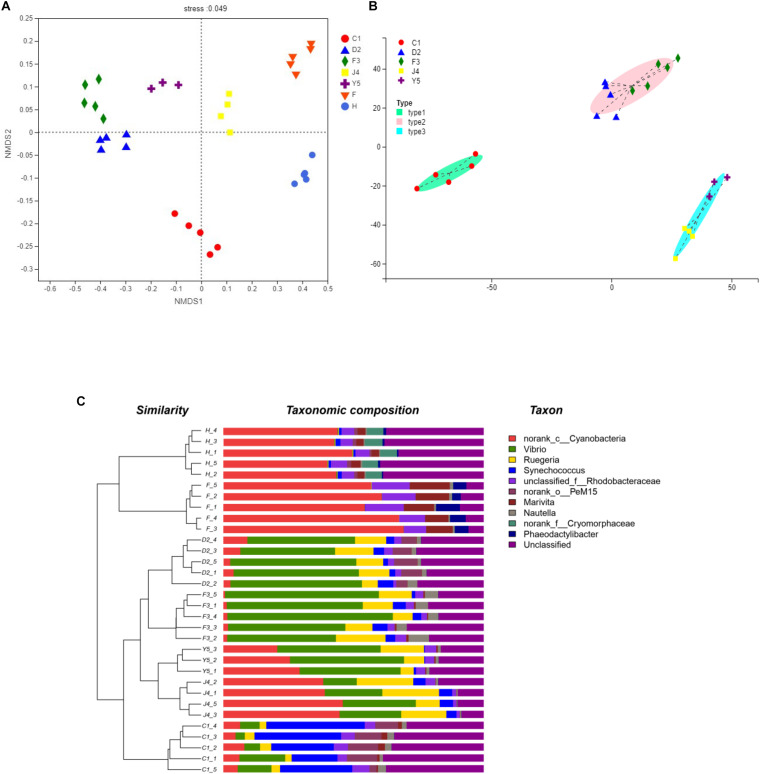
Beta diversity analysis. NMDS analysis **(A)**, Typing analysis **(B)** at the genus level was based on the weighted UniFrac distances. A UPGMA tree **(C)** (based on the weighted UniFrac distances) and a genus-level relative abundance map are shown on the left and right, respectively. C1, one-spiral whorl stage; D2, two-spiral whorl stage; F3, three-spiral whorl stage; J4, four-spiral whorl stage (competent larvae); Y5, postlarval stage after 3 days of metamorphosis; F, food; H, water.

The beta diversity of the microbiota community may suggest that structure of microbiota changes with development. NMDS analysis shown in Figure 4A and all the stages were clustered well. Meanwhile, typing analysis showed that stages D2 and F3 belonged to the same type, and stages J4 and Y5 belonged to the same type, while stage C1 alone belonged to a single type (Figure 4B). Meanwhile, unweighted pair group method with arithmetic mean (UPGMA) clustering showed that all the individual samples were clustered into groups according to the culture stage (Figure 4C). Groups D2 and F3 were clustered into the same branch, and groups J4 and Y5 were clustered into the same branch, while group C1 alone was clustered into a single branch, which was consistent with the results of the NMDS and the typing analysis. Adonis and ANOSIM showed that the symbiotic microbiota populations have significantly difference between any two of the compared stages (*P*-value < 0.05) (Table 2).

**TABLE 2:** Adonis and ANOSIM tests of significant differences between the five culture stages.

Group	Adonis			ANOSIM	
	df	R2	*P*-value	*R*	*P*-value
Total	6	0.94883	0.001	0.9887	0.001
C1/D2/F3/J4/Y5	4	0.882377	0.001	0.9634	0.001
C1/D2	1	0.834953	0.005	1	0.011
C1/F3	1	0.865953	0.008	1	0.006
C1/J4	1	0.738655	0.004	1	0.013
C1/Y5	1	0.809489	0.023	1	0.021
D2/F3	1	0.483972	0.012	0.812	0.011
D2/J4	1	0.895553	0.005	1	0.009
D2/Y5	1	0.757638	0.016	1	0.017
F3/J4	1	0.882483	0.013	1	0.011
F3/Y5	1	0.737072	0.016	1	0.018
J4/Y5	1	0.760203	0.022	1	0.034

*The Adonis test is a non-parametric MANOVA. R2 represents the degree of variation between groups that was explained. The credibility of this test is high (P-value < 0.05). ANOSIM tests whether the symbiotic microbiota varied at different culture stages. The R-value was calculated based on the Bray–Curtis dissimilarity (P-value < 0.05). The closer the R value was to 1, the larger the difference between the groups compared to the difference within the groups (P-value < 0.05). C1, one-spiral whorl stage; D2, two-spiral whorl stage; F3, three-spiral whorl stage; J4, four-spiral whorl stage (competent larvae); and Y5, postlarval stage after 3 days of settling.*

### Changes in the Dominant Bacterial Community With Host Development

**FIGURE 5:**
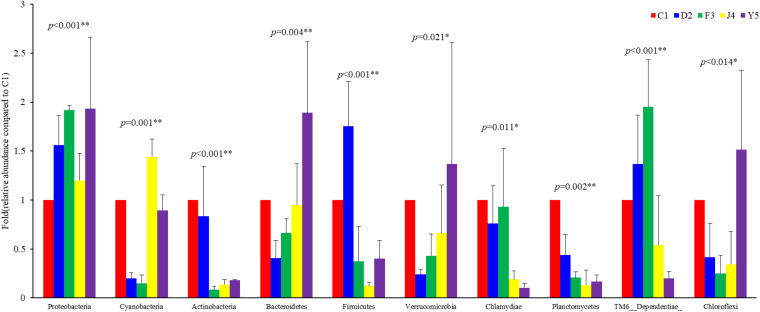
Abundances of the 10 most abundant phyla at different culture stages. The relative abundance of each phylum at the five culture stages is shown. The abundance at C1 was assigned a value of 1, and the relative abundances at other stages were calculated relative to this abundance. Asterisks indicate significant differences between groups by PERMANOVA (*P* < 0.05). Two asterisks indicate extremely significant differences between the groups (*P* < 0.01). C1, one-spiral whorl stage; D2, two-spiral whorl stage; F3, three-spiral whorl stage; J4, four-spiral whorl stage (competent larvae); Y5, postlarval stage after 3 days of metamorphosis.

Among the top 10 phyla, the Kruskal–Wallis *H* test demonstrated that the abundances of Proteobacteria, Cyanobacteria, Actinobacteria, Bacteroidetes, Firmicutes, Verrucomicrobia, Chlamydiae, Planctomycetes, TM6_Dependentiae, and Chloroflexi changed significantly between different culture stages (*P*-value < 0.05) (Figure 5). Proteobacteria, the most dominant phylum, showed an increasing trend before the J4 stage and became dramatically depleted in the J4 stage. The abundances of Verrucomicrobia and Bacteroidetes increased from the D2 stage, while the abundances of Chlamydiae and Planctomycetes decreased with host development.

**FIGURE 6:**
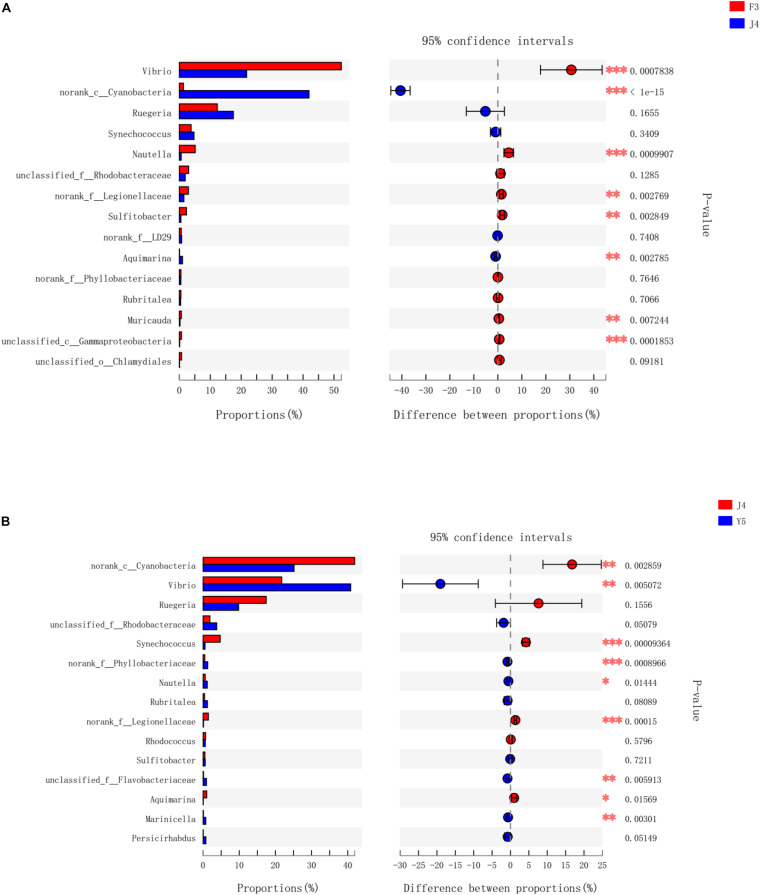
Bacterial taxa differentially represented between the three groups (F3, J4, and Y5) as determined by LEfSe using default parameters. F3 vs J4 **(A)**, J4 vs Y5 **(B)**. The asterisks indicate significant differences between the groups (*P* < 0.05). Two asterisks indicate extremely significant differences between the groups (P < 0.01). F3: three-spiral whorl stage, J4: four-spiral whorl stage (competent larvae) and Y5: postlarval stage after 3 days of metamorphosis.

To further investigate the different genera in the F3, J4, and Y5 stages, we conducted pairwise comparisons of relative abundances in the three stages using Student’s *t*-test. The results showed that the relative abundances of *Vibrio, norank_c_Cyanobacteria, Nautella, norank_f_legionellaceae, Aquimarina*, and *Muricauda* were significantly different in both F3 vs. J4 and J4 vs. Y5 and the relative abundance of *Vibrio* in F3 and Y5 was significantly higher than that in J4. In contrast, norank_c_*Cyanobacteria*, *Sulfitobacter*, and *Gammaproteobacteria* showed significant differences between F3 and J4, and *Synechococcus, norank_f_Phyllobacteriaceae and unclassified_f_Flavobacteriaceae* showed significant differences in J4 vs. Y5 (Figure 6).

### Functional Prediction for the Symbiotic Microbiota

**FIGURE 7:**
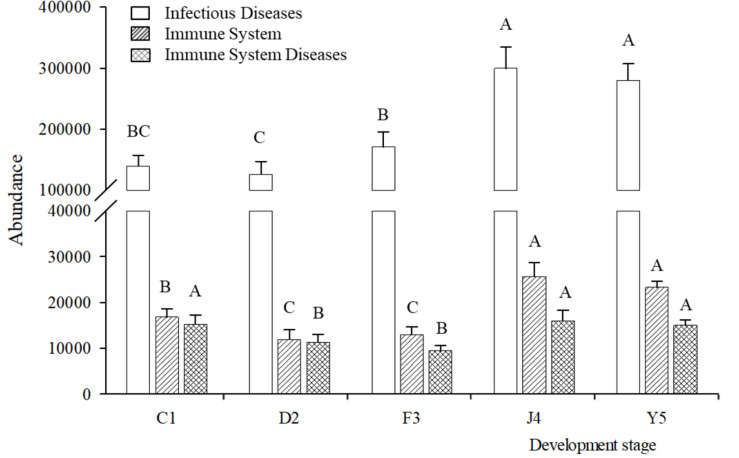
Relative abundances of predicted functions. Relative abundances of immune-related KOs at KEGG level 2 in different development stages. Different upper-case letters within each bar indicate significant differences (*P* < 0.05). C1, one-spiral whorl stage; D2, two-spiral whorl stage; F3, three-spiral whorl stage; J4, four-spiral whorl stage (competent larvae); Y5, postlarval stage after 3 days of metamorphosis.

Changes in the putative functions of the symbiotic microbiota in *R. venosa* were analyzed by predicting metagenomes using PICRUSt, and the results showed that the symbiotic microbiota was mainly enriched in functions related to transporters, ATP-binding cassette (ABC) transporters, purine metabolism, ribosomes, peptidases, oxidative phosphorylation, bacterial motility proteins, porphyrin and chlorophyll metabolism, pyrimidine metabolism and amino acid-related enzymes (Supplementary Figure S1). We chose three KOs (KEGG Orthologs) at KEGG level 2 that are related to immune function (infectious diseases, immune system, immune system diseases) and analyzed their relative abundances among the five development stages. We found that all three KOs had high abundances in the J4 and Y5 stages, and their abundances increased with host development, peaking in the J4 stage (Figure 7).

### Host Immune Response

**FIGURE 8:**
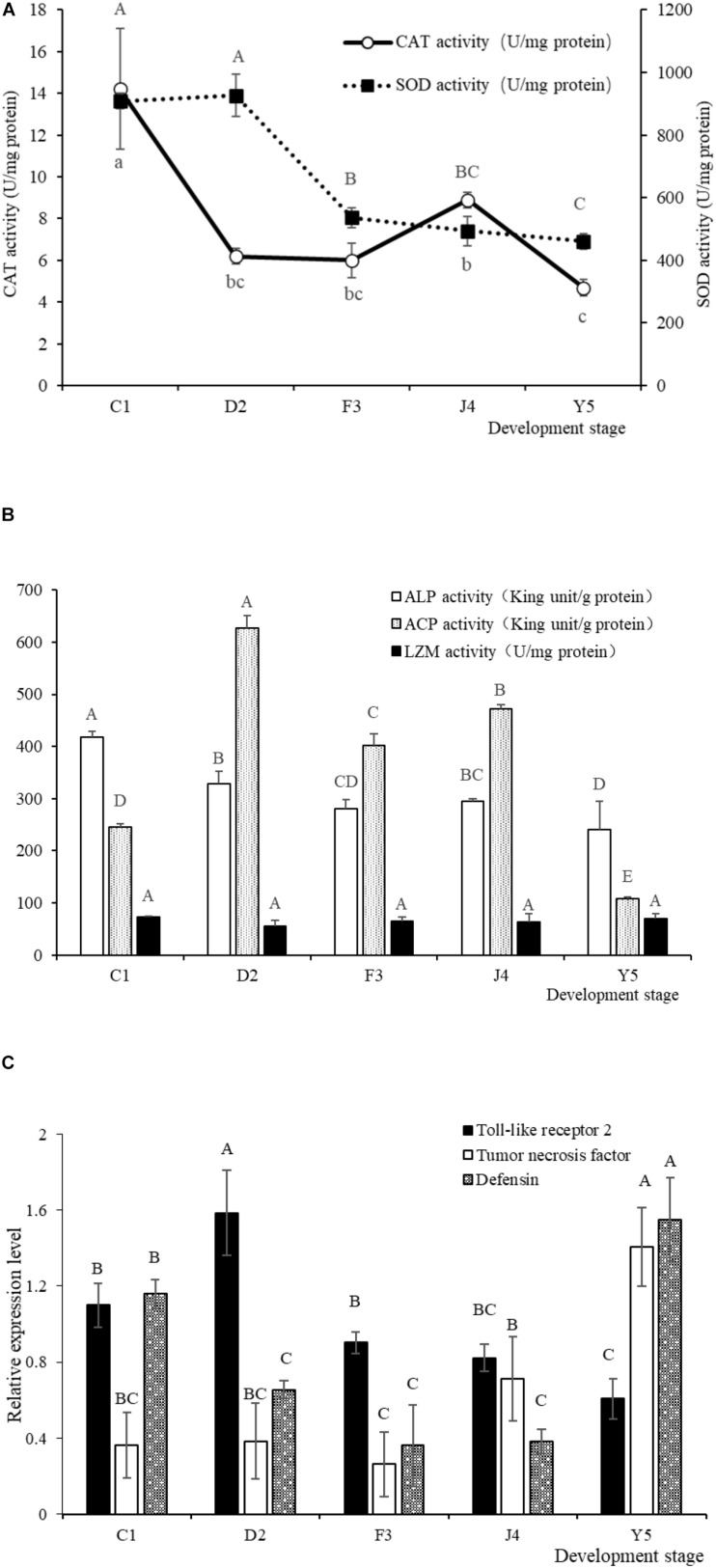
SOD and CAT activities **(A)** and LSZ, ACP and ALP activities **(B)** in larvae of *R. venosa*. Relative mRNA expression of Toll-like receptor 2, tumor necrosis factor and defensin **(C)**. Values are expressed as the mean ± SD (*n* = 5). Different upper-case letters within each bar indicate significant differences (*P* < 0.05). C1, one-spiral whorl stage; D2, two-spiral whorl stage; F3, three-spiral whorl stage; J4, four-spiral whorl stage (competent larva); Y5, postlarval stage after 3 days of metamorphosis.

The SOD and CAT activities in the larvae exhibited a decreasing trend with the development of *R. venosa* (Figure 8A) and reached their lowest levels at the Y5 stage (*P* < 0.05). The ALP and ACP activities were also at their lowest levels at the Y5 stage, while the LSZ activity did not change significantly during the early development stages (*P* < 0.05) (Figure 8B).

The relative expression of Toll-like receptor 2, tumor necrosis factor and defensin significantly changed during host development (Figure 8C). The relative expression of Toll-like receptor 2 peaked at the D2 stage, after which the expression of this gene decreased significantly with host development, becoming lowest at the Y5 stage. The relative expression of tumor necrosis factor increased with host development and peaked at the Y5 stage, which is similar to the observed trend for defensin.

## Discussion

Increased attention is being paid to the relationship between aquatic hosts and their symbiotic microbiota. Previous researches have indicated that symbiotic microbiota is important for the maturation of immune system (Mazmanian et al., 2005; Edelman et al., 2008), so there may be some changes in the symbiotic microbiota during host development. Present results showed that significant shifts in the structure of symbiotic microbiota and immune response of *R. venosa* occurred at early development stages, which may reflect a substantial change in the physiological functions of the larvae.

The core microbiota consists of the OTUs shared among all samples (Turnbaugh et al., 2009). In our study, 102 OTUs were shared by all samples from the host, accounting for 5.85% of the total OTUs (Figure 2). The core microbiota could reflect the impact of diet, development, stocking density and environment on microbiota (Wong et al., 2013) and is usually determined based on the genetic background. We found that most of the shared OTUs belonged to Proteobacteria and Cyanobacteria, which were also the dominant phyla in *R. venosa* in this study. Proteobacteria are commonly found in the digestive tract of adult *R. venosa* (Yang et al., 2019), Pacific white shrimp (Zeng et al., 2017) and gibel carp (Li et al., 2017) and seem to be the dominant phylum among aquacultured animals, which may result from the widespread distribution of Proteobacteria in environmental water and the ability of these bacteria to interact with host. However, Cyanobacteria are not abundant in adult whelks (Song et al., 2018; Yang et al., 2019) and have a low abundance (less than 0.01%) in most aquaculture animals (Dhanasiri et al., 2011; Rungrassamee et al., 2014; Li et al., 2017), while they were detected at a high abundance (4.5–37.0%) in this study, which might be associated with the water environment and feed because Cyanobacteria were also the dominant phylum in water and feed samples (Figure 1A).

A previous study has indicated that neutral processes are able to dramatically affect the gut microbiota during development in zebrafish (Stephens et al., 2015). Li et al. (2017) also indicated that the structure of gut microbiota in gibel carp mainly depended on by host-associated processes instead of stochastic processes, and they found that the diversity of gut microbiota community increased with developing, which may be related to the morphological differentiation of the gastrointestinal tract (Li, 2007). In present study, the diversity of symbiotic microbiota showed a decreasing trend during the development stages, while the richness indices showed no significant change, which suggests that the role of the dominant population becomes increasingly prominent as the host develops. Moreover, UPGMA clustering, NMDS and typing analysis also showed differences in the symbiotic microbiota between development stages. The microbiota compositions of stages D2 and F3 are highly similar, while those of J4 and Y5 belong to the same type. Metamorphosis occurs in the late phase of the J4 stage, so there should be a considerable difference in the symbiotic microbiota between the J4 and Y5 stages. However, in our study, significant changes were observed earlier than expected, which may suggest that a series of changes in the internal physiological functions of the host during metamorphosis precede the changes in apparent features, including the further development of the digestive and immune system. This shift in the symbiotic microbiota in *R. venosa* occurs because the conditions of host (e.g., physicochemical environments, niche availability, and microbial interactions) change dramatically during host development.

Many studies have reported that the balance of the symbiotic microbiota could make difference on immune system, especially in invertebrates lacking specific immune system, which is crucial for disease resistance (Gómez and Balcázar, 2008; Jaenike et al., 2010). Therefore, changes in the abundance of some pathogenic bacteria may reflect changes in the immune function of the host. Chlamydiae is an obligate intracellular bacterium that can cause many diseases in the host (Moulder, 1991). In the present study, the abundance of Chlamydiae decreased significantly with host development, which may indicate the strengthening of host immune function. Additionally, the genus *Vibrio*, which is widely distributed in aquaculture, contains many pathogenic species, such as *Vibrio anguillarum*, *Vibrio ordalii*, and *Vibrio parahaemolyticus*, which can cause disease in fish, shrimp and shellfish. Previous studies have indicated that *V. anguillarum* has a noticeable impact on the phosphatase activity of *Cyclina sinensis* and a significant stimulatory effect on the immune system of clams (Song et al., 2010). We found that the abundance of *Vibrio* decreased dramatically in the J4 stage in the present study, which may be another indicator of changes in the immune system that occur during metamorphosis in the host. All three KOs predicted by PICRUSt are related to immune function, namely, infectious diseases, immune system and immune system diseases, had significantly higher abundances in the J4 and Y5 stages than in the other stages, indicating the involvement of many microbes in the host immune system. However, the applicability of PICRUSt prediction in *R. venosa* needs further validation, although PICRUSt prediction in humans shows consistency across all body tissues.

There is no specific immune system in mollusks, which mainly rely on non-specific immunity to prevent infections and maintain good health. The phagocytic process is the first line of internal defense in the body (Janeway and Medzhitov, 2002), and mollusk phagocytes can completely degrade exogenous bacteria via the activities of LSZ, ACP, and ALP (Canicatti, 1990; Ellis, 1999). Therefore, the activity of these immune-related enzymes can reflect the body’s immune status. ACP and ALP are important hydrolases that participate in immune defense (Xiao et al., 2007). ACP is widely distributed in animal tissues and can destroy foreign bodies through hydrolysis to prevent pathogenic infection (Cheng, 1978; Lackie, 1980). ALP is mainly located on the plasma membranes of cells and is an important metabolic regulatory enzyme in animals. ALP is also related to calcium absorption, membrane absorption and transport, and keratin secretion in shellfish. Moreover, ALP as a significant component of animal lysosomal enzymes, plays an important role in the immune response (Xie et al., 2000). In our study, ACP activity was significantly decreased in the Y5 stage, while ALP and LZM activities exhibited no substantial change. CAT and SOD are the primary components of the antioxidant defense system of organisms, and the activities of these enzymes could indicate the ability of body to resist oxidative stress though reducing the H_2_O_2_ and O_2_^–^ content and indirectly reflect the immunity levels (Bao et al., 2017). The activities of CAT and SOD were dramatically decreased during the early development of *R. venosa*, which might be related to the decreases in the abundances of pathogens, such as Chlamydiae and *Vibrio*. On the other hand, ALP, CAT and SOD all exhibited high activities in the C1 stage, which may be because larvae hatching from the oocysts first enter the water environment with an abundance of microorganisms and initial colonization by the symbiotic microbiota in larvae stimulates the immune system of the host.

Previous research has indicated that Toll-like receptor 2 can identify the capsular polysaccharide antigens of commensal bacteria, stimulating the innate immune response (Edelman et al., 2008). We found that the expression of the Toll-like receptor 2 gene was not significantly increased in the Y5 stage, which was not in line with the results in previous study (Song et al., 2016b), and we speculated that this difference is related to the differences in the composition of the sampled microbiota in different environments. Furthermore, tumor necrosis factor and defensin are two other important genes related to the symbiotic microbiota in the host (Jordans and Misra, 2012; Yuzhakova et al., 2016). Tumor necrosis factor exhibited a high expression level in the Y5 stage, which may suggest that this gene is related to the shift in symbiotic microbiota during metamorphosis. On the other hand, defensin also exhibited relatively high expression in the C1 stage, which indicates that defensin is associated with not only the shift in symbiotic microbiota during metamorphosis but also the initial colonization in host by the symbiotic microbiota.

The host and symbiotic microbiota are components of an indivisible whole that interact with each other. On the one hand, the development of immune system in host affects the structure of the symbiotic microbiota. On the other hand, the colonization by and shift in the symbiotic microbiota stimulate the development of the immune system. Our present study comprehensively examined the symbiotic microbiota and the immune response in *R. venosa* during the early development stage and investigated the relationship between the symbiotic microbiota and the immune system of the host. We found that dramatic changes in the symbiotic microbiota and the immune response mainly occurred in the initially hatched veliger (C1), competent larval (J4) and postlarval (Y5) stages, during which the hosts experienced substantial environmental changes or changes in physiological structure and function. These findings expand our knowledge of the stage-specific symbiotic microbiota in *R. venosa* and the close association between symbiotic microbiota and immune system in mollusks. However, additional studies are needed to explore the specific relationship between microbiota and immune system during the early development stages, especially during metamorphosis.

## Data Availability Statement

The datasets generated for this study can be found in the SRA under accession number PRJNA574861.

## Author Contributions

TZ conceived and designed the experiments. M-JY conducted the experiments. M-JY and HS analyzed the data. Z-LY, ZH, CZ, and X-LW contributed reagents, materials, and analytical tools. M-JY wrote the manuscript.

## Conflict of Interest

The authors declare that the research was conducted in the absence of any commercial or financial relationships that could be construed as a potential conflict of interest.

## Footnotes

1 http://drive5.com/uparse/

2 http://drive5.com/usearch/manual/dereplication.html

3 http://drive5.com/usearch/manual/singletons.html

4 https://drive5.com/usearch/manual/chimeras.html

5 http://www.arb-silva.de

6 http://rdp.cme.msu.edu/

7 www.majorbio.com

8 http://www.mothur.org/wiki

9 http://bioinformatics.psb.ugent.be/webtools/Venn/

10 https://cran.r-project.org/web/packages/vegan/

11 http://qiime.org/index.html

12 http://huttenhower.sph.harvard.edu/galaxy/

## Supplementary Material

FIGURE S1 Relative abundances of predicted functions. KOs at KEGG level 3 are listed according to the relative average abundances. C1, one-spiral whorl stage; D2, two-spiral whorl stage; F3, three-spiral whorl stage; J4, four-spiral whorl stage (competent larvae); Y5, postlarval stage after 3 days of metamorphosis.

TABLE S1 Immune-related genes analyzed in the present work and primer sequences used for qPCR.
