# Supplementary figures and images for: Changes in Symbiotic Microbiota and Immune Responses in Early Development Stages of Rapana venosa (Valenciennes, 1846) Provide Insights Into Immune System Development in Gastropods
Source: Front Microbiol. 2020 Jun 16;11:1265. doi: 10.3389/fmicb.2020.01265 (PMC7308808; doi:10.3389/fmicb.2020.01265)

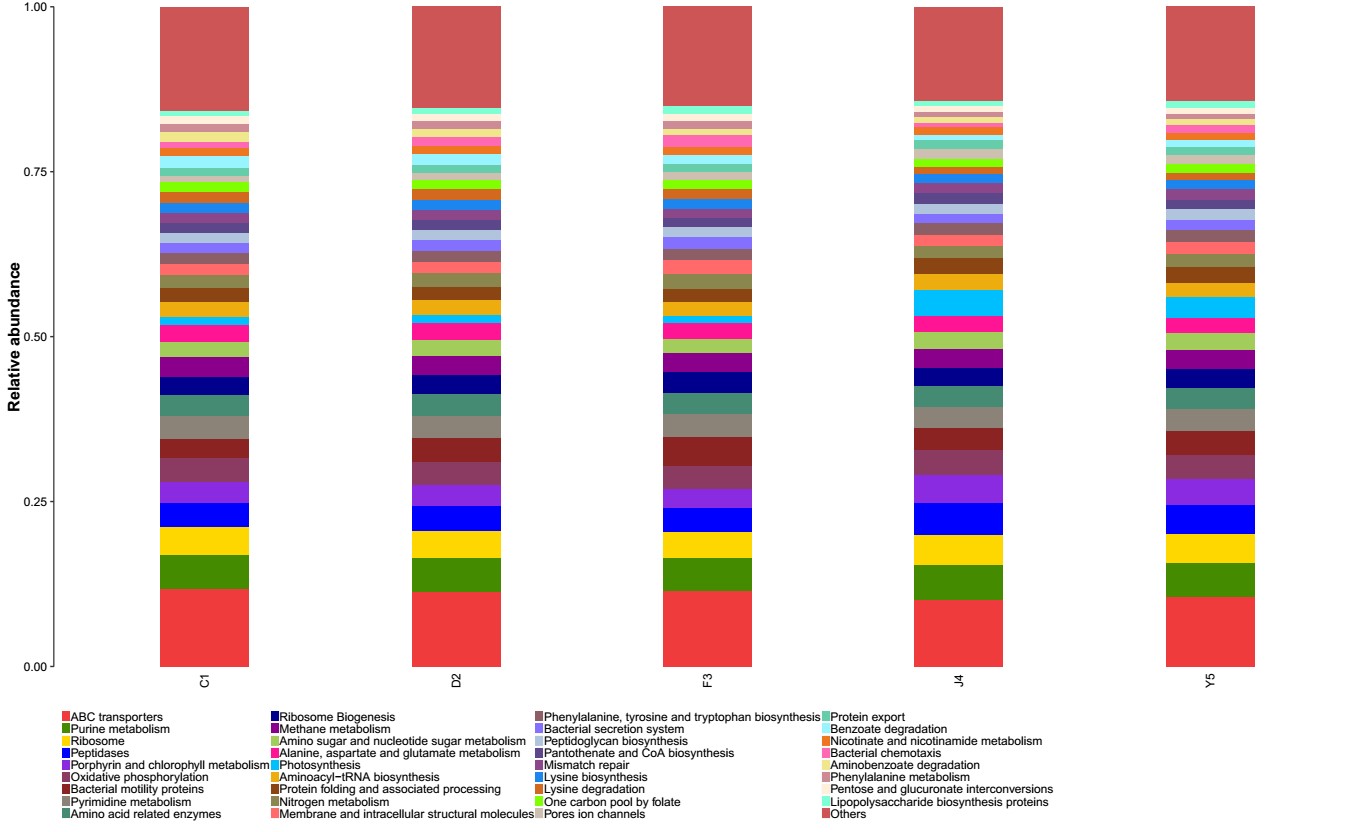

Supplement: FIGURE S1 — Relative abundances of predicted functions. KOs at KEGG level 3 are listed according to the relative average abundances. C1, one-spiral whorl stage; D2, two-spiral whorl stage; F3, three-spiral whorl stage; J4, four-spiral whorl stage (competent larvae); Y5, postlarval stage after 3 days of metamorphosis. [file Image_1.JPEG]
