# Supplementary material for: Changes in Symbiotic Microbiota and Immune Responses in Early Development Stages of Rapana venosa (Valenciennes, 1846) Provide Insights Into Immune System Development in Gastropods
Source: Front Microbiol. 2020 Jun 16;11:1265. doi: 10.3389/fmicb.2020.01265 (PMC7308808; doi:10.3389/fmicb.2020.01265)
Supplement: TABLE S1 — Immune-related genes analyzed in the present work and primer sequences used for qPCR. [file Table_1.docx]

**Table S1. Immune-related genes analyzed in the present work and primer sequences used for qPCR.**

| **Gene** | **Primer** | **Sequence (5’-3’)** |
| --- | --- | --- |
| Defensin | De-F | GGCTTCAGTCGTCAGGACAATGC |
|  | De-R | CAGCAGAGGCCGAGTGAAGAATTG |
| Tumor necrosis factor | Tu-F | TCTGTGCTGCCCTCTTTGTC |
|  | Tu-R | GCCCTCGCTGTACTTGTCG |
| toll-like receptor 2 | To-F | CCTCCTGCTCCACAACCTCTCC |
|  | To-R | GTGCTTGTCCTCGGTGCTGATG |
| 60S ribosomal protein L28 | RL28-F | CGTGCGTAACATCACCAAGA |
|  | RL28-R | CACCACAGCTACCACACATT |
